# Amplification of TGFβ Induced ITGB6 Gene Transcription May Promote Pulmonary Fibrosis

**DOI:** 10.1371/journal.pone.0158047

**Published:** 2016-08-05

**Authors:** Amanda L. Tatler, Amanda T. Goodwin, Olumide Gbolahan, Gauri Saini, Joanne Porte, Alison E. John, Rachel L. Clifford, Shelia M. Violette, Paul H. Weinreb, Helen Parfrey, Paul J. Wolters, Jack Gauldie, Martin Kolb, Gisli Jenkins

**Affiliations:** 1 Division of Respiratory Medicine–City, University of Nottingham, Nottingham, United Kingdom; 2 Biogen, Cambridge, Massachusetts, United States of America; 3 Department of Pathology, University of Cambridge, Cambridge, United Kingdom; 4 School of Medicine, University of California San Francisco, San Francisco, California, United States of America; 5 Department of Pathology and Molecular Medicine, McMaster University, Hamilton, Canada; University of Pittsburgh, UNITED STATES

## Abstract

Idiopathic pulmonary fibrosis (IPF) is a devastating, progressive disease with poor survival rates and limited treatment options. Upregulation of αvβ6 integrins within the alveolar epithelial cells is a characteristic feature of IPF and correlates with poor patient survival. The pro-fibrotic cytokine TGFβ1 can upregulate αvβ6 integrin expression but the molecular mechanisms driving this effect have not previously been elucidated. We confirm that stimulation with exogenous TGFβ1 increases expression of the integrin β6 subunit gene (*ITGB6)* and αvβ6 integrin cell surface expression in a time- and concentration-dependent manner. TGFβ1-induced *ITGB6* expression occurs via transcriptional activation of the *ITGB6* gene, but does not result from effects on *ITGB6* mRNA stability. Basal expression of *ITGB6* in, and αvβ6 integrins on, lung epithelial cells occurs via homeostatic αvβ6-mediated TGFβ1 activation in the absence of exogenous stimulation, and can be amplified by TGFβ1 activation. Fundamentally, we show for the first time that TGFβ1-induced *ITGB6* expression occurs via canonical Smad signalling since dominant negative constructs directed against Smad3 and 4 inhibit *ITGB6* transcriptional activity. Furthermore, disruption of a Smad binding site at -798 in the *ITGB6* promoter abolishes TGFβ1-induced *ITGB6* transcriptional activity. Using chromatin immunoprecipitation we demonstrate that TGFβ1 stimulation of lung epithelial cells results in direct binding of Smad3, and Smad4, to the *ITGB6* gene promoter within this region. Finally, using an adenoviral TGFβ1 over-expression model of pulmonary fibrosis we demonstrate that Smad3 is crucial for TGFβ1-induced αvβ6 integrin expression within the alveolar epithelium *in vivo*. Together, these data confirm that a homeostatic, autocrine loop of αvβ6 integrin activated TGFβ1-induced *ITGB6* gene expression regulates epithelial basal αvβ6 integrin expression, and demonstrates that this occurs via Smad-dependent transcriptional regulation at a single Smad binding site in the promoter of the β6 subunit gene. Active TGFβ1 amplifies this pathway both *in vitro* and *in vivo*, which may promote fibrosis.

## Introduction

Idiopathic pulmonary fibrosis (IPF) is a progressive, fibrotic lung disease of unknown aetiology and increasing incidence [1]. It is characterised by the pathological deposition of fibrous matrix, particularly collagen, within the lung parenchyma, leading to rapidly decreasing lung function and ultimately respiratory failure. The survival of IPF patients is poor with 5-year survival rates worse than most cancers [2] and there are currently limited treatment options available due, in part, to our limited understanding of the mechanisms underlying the disease.

Activation of extracellular stores of the potently pro-fibrotic cytokine Transforming Growth Factor-β1 (TGFβ1) is a fundamental process in fibrogenesis in the lung and many other organs. Numerous mechanisms of TGFβ1 activation have been described in many cell types [3] but activation via cell surface integrin receptors has been shown to be important *in vivo*, particularly in the context of fibrogenesis [4, 5]. Importantly, animal models of fibrosis have shown that activation of TGFβ1 by αvβ6 integrins is a central process in disease pathogenesis in a number of organs, since loss of αvβ6 expression, or blockade of αvβ6 functions, interrupts fibrogenesis [6–9]. In the lung epithelium αvβ6 integrins activate TGFβ1 following G-protein signalling in response to mediators associated with cell injury and repair [10–12].

αvβ6 integrins are exclusively expressed by epithelial cells. In normal, healthy tissues αvβ6 expression is tightly regulated, however, in response to injury their expression is dramatically upregulated. Increased expression of αvβ6 integrins within epithelial cells is a common feature of fibrosis in many organ systems including in the lungs of idiopathic pulmonary fibrosis (IPF) patients [6, 9, 10] and in animal models of pulmonary fibrosis [13]. Fundamentally, increased expression levels of αvβ6 integrins correlate with poorer survival in patients suffering from idiopathic pulmonary fibrosis [14], and increased levels of mRNA for the β6 subunit gene (*ITGB6*) correlate with increasing severity in liver fibrosis patients [15], suggesting upregulation of αvβ6 integrins may be an important process in fibrotic diseases. The molecular mechanisms responsible for basal αvβ6 integrin expression and upregulation following injury have not been thoroughly delineated.

Early studies demonstrated that TGFβ1 increases mRNA levels of the β6 integrin subunit (*ITGB6)* in guinea pig epithelial cells *in vitro* [16]. Furthermore, a positive-feedback loop of αvβ6-mediated TGFβ1 activation promoting enhanced αvβ6 expression in the lung epithelium has been proposed but not confirmed [17]. TGFβ1-mediated upregulation of *ITGB6* and αvβ6 integrins in human lung epithelial cells may involve either Smad-dependent or Smad-independent pathways. The aims of this study were to investigate the signalling pathways involved in regulation of epithelial αvβ6 integrins *in vitro* and *in vivo*.

## Materials and Methods

### Cell Culture

*In vitro* experiments were performed on immortalised human bronchial epithelial cells (iHBECs; gift from Prof. Jerry Shay, University of Texas, USA). iHBECs were selected for the technical advantages of performing complex molecular assays such as chromatin immunoprecipitations and transfections in a continuously dividing cell line. These cells retain many of the properties of primary epithelial cells, including the ability to differentiate in to ciliated, basal and mucous producing epithelial cells, and are one of the only immortalised epithelial cell lines to retain their expression of αvβ6 integrins *in vitro*. iHBECs were cultured in keratinocyte serum free media (KSFM; Gibco, UK) supplemented with 25μg/ml bovine pituitary extract (Gibco, UK), 0.2ng/ml recombinant epithelial growth factor (Gibco, UK), 250ng/ml puromycin (Sigma-Aldrich, UK) and 25μg/ml G418 (Sigma-Aldrich, UK). Cells were growth arrested in unsupplemented KSFM for 24 hours prior to experimentation.

Small airway epithelial cells (SAECs) were used to confirm some of the key findings obtained from experiments using iHBECs. SAECs were purchased from Lonza, UK and were used at passage 2–3. They were cultured in small airway growth media (SAGM; Lonza, UK) containing the supplied supplements (bovine pituitary extract, hydrocortisone, epidermal growth factor, epinephrine, transferrin, insulin, retinoic acid, triiodothyronine, gentamycin and bovine serum albumin). Cells were growth arrested in unsupplemented SAGM for 24 hours prior to experimentation.

### Quantitative Polymerase Chain Reaction (QPCR)

Gene expression changes at the mRNA level were assessed by QPCR using a MXPro3000 thermocycler (Stratagene, USA) and Kapa SYBR Fast taq (Kapa Biosystems, Japan) as previously described (Tatler et al 2011). Briefly, total cell RNA was isolated using a Nucleospin RNA II isolation kit (Macharey Nagel, UK) and reverse transcribed in to complimentary DNA (cDNA) using murine Moloney leukaemia virus (Promega, UK). The resulting cDNA was subjected to QPCR analysis using the following primer sequences and a 60°C annealing temperature: *ITGB6* sense 5’-AAACGGGAACCAATCCTCTGT, antisense 5’-GCTTCTCCCTGTGCTTGTAGGT-3’; β-2-microglobulin (*B2M)* sense 5’-AATCCAAATGCGGCATCT-3’, antisense 5’-GAGTATGCCTGCCGTGTG-3’. Amplification of a single DNA product was confirmed by melting curve analysis and expression levels were calculated using the Ct equation.

### Flow Cytometry

Expression of αvβ6 integrins on the surface of epithelial cells was determined by flow cytometry as previously described (Xu et al 2009). Non-specific binding of anti-αvβ6 antibodies (clone 6.3G9; Biogen Idec, USA) was blocked by incubating iHBECs (100,000 cells) with 5% goat serum (Sigma-Aldrich, UK) for 20 minutes. The cells were then labelled with 10μg anti-αvβ6 antibody in PBS for 20 minutes and an anti-mouse phycoerythrin conjugated secondary antibody (1:200 dilution; New England Biolabs, UK) for 20 minutes. Surface expression was analysed in 10,000 cells using a FacsDIVA flow cytometer (BD, UK) and data was analysed using FlowJo version 10.1 (FlowJo, USA).

### mRNA Stability Assay

Stability of *ITGB6* mRNA was determined using the inhibitor of transcription actinomycin D. Following treatment with 2ng/ml TGFβ1 (R and D Systems, UK) for 4 hours iHBECs were treated with 5μg/ml actinomycin D (Sigma-Aldrich, UK) to inhibit further transcription. mRNA was collected at increasing time points over 24 hours prior to expression of *ITGB6* being determined by QPCR.

### Generation of pGL3-*ITGB6* Promoter Reporter Construct

The 1.1kb insert was excised from pGL2-*ITGB6* [18] using *XhoI* and *BamHI* restriction enzymes (both from New England Biolabs, UK) and ligated into the pGL3 vector (Promega, UK) using T4 ligase (New England Biolabs, UK) according to the manufacturer’s instructions.

### Reporter Construct Transfections

Transient transfections were performed using Transfast (Promega, UK) transfection reagent using 0.75μg reporter plasmid DNA with 7.5ng renilla luciferase DNA at a 1:2 DNA: Transfast ratio. Briefly, cells were seeded at 2 x 10^5^ cells/ml then cultured for 8 hours in supplemented KSFM prior to transfection overnight in unsupplemented KSFM. The following day cells were stimulated as required for the experiment. Luciferase activity was determined using the dual luciferase reporter assay system (Promega, UK).

### Dominant Negative Smad2 and Smad3 Transfections

dnSmad2 and dnSmad3 expression constructs were a kind gift from Prof. Sturrock, University of Utah, USA. dnSmad4 was obtained from Addgene (Plasmid 14040). Transfection was performed using 0.1μg dominant negative plasmid DNA and a 1:3 DNA:Transfast ratio in unsupplemented KSFM. *In vitro* experiments were performed 24 hours after transfection to allow expression of dominant negative proteins.

### Site Directed Mutagenesis (SDM)

Site directed mutagenesis (SDM) was performed using the QuickChange II Site Directed Mutagenesis kit (Agilent, UK) according to the manufacturer’s instructions. 5ng pGL3-*ITGB6* promoter construct was used as a DNA template for the reaction. The Smad binding sites located at -864, -866 and -798 from the transcription start site (TSS) were mutated from CAGA to TACA using SDM primers designed on www.genomics.agilent.com/primerdesign. All constructs were sequenced prior to use in transfection experiments to confirm the presence of the correct mutation.

### Chromatin immunoprecipitation (ChIP)

Chromatin immunoprecipitation (ChIP) allowed the binding of Smad proteins to the *ITGB6* promoter to be determined. The ChIP-IT Express kit (Active Motif, UK) was used to assess binding in cultured cells as previously described for airway smooth muscle cells [19]. Briefly, cells were fixed with 1% formaldehyde for 5 minutes and lysed prior to shearing of the cellular chromatin by sonication using an Epishear sonicator (Active Motif, UK). 25μg total chromatin was immunoprecipitated with 10μg Smad3 antibody (Abcam AB28379) and Protein G magnetic beads. Proteins were then digested with proteinase K and the immunoprecipitated DNA subjected to QPCR analysis for *ITGB6* expression.

ChIP on human lung tissue was performed as previously described [20]. Briefly, a single cell suspension was generated by passing the lung tissue through a 100μm cell strainer. The cellular chromatin was then cross-linked in 1% formaldehyde for 5 minutes. The crosslinking was reversed with glycine, the cells lysed in 10% SDS, and then the chromatin was sheared by sonication using an Epishear sonicator. 100μg of total chromatin was subjected to immunoprecipitation with 10μg of Smad3 antibody (Abcam AB28379) overnight. Antibody-bound DNA was extracted using Zysorbin and subjected to QPCR analysis for *ITGB6* expression.

*ITGB6* DNA immunoprecipitated in all ChIP experiments was detected using the following primers: *ITGB6* promoter -934 to -753 sense 5’-CATGCTTACCCAGGAATGCT-3’ and anti-sense 5’-ACACCCTGGGGGAAAAATAC-3’

### *In vivo* Adenoviral TGFβ1 Model of Pulmonary Fibrosis

All animal studies using adenoviral TGFβ1 were approved by the Animal Research Ethics Board of McMaster University, Canada and conducted in accordance with the guidelines of the Canadian Council of Animal Care. Animals received free access to food and water at all times. Sprague Dawley rats or Smad3^-/-^ mice [21] and wild-type mice (aged 5–6 weeks) were treated with either an adenovirus encoding active TGFβ1 or a control adenovirus as previously described [21]. The lungs were excised after 21 days, insufflated with formalin and paraffin wax embedded prior to immunohistochemical analysis.

### Immunohistochemistry

5μm thick sections of paraffin wax embedded lung tissue of murine and rat origin was subjected to immunohistochemistry to evaluate expression levels of the αvβ6 integrin as previously described [10]. An antibody directed against murine/rat αvβ6 (clone ch2.A1) was kindly provided by Biogen Idec, USA. Briefly, tissue was dewaxed in xylene and rehydrated in decreasing concentrations of ethanol and then boiled in 10mM citrate buffer to retrieve endogenous antigens. Following blocking in 5% goat serum the sections were incubated in αvβ6 antibody (0.5μg/ml) overnight. Staining was visualised using DAB.

### αvβ6 Immunohistochemistry Quantification

Immunostaining for αvβ6 integrins was quantified using an operator-dependent semi-quantitative method as previously described [13]. The percentage of epithelial cells expressing αvβ6 across the tissue section was calculated by assessing five random fields captured at x40 magnification using a Nikon Eclipse 90i microscope and NIS Elements image analysis software. Each image was overlaid with a grid containing 192 squares of 13μm^2^. The number of squares containing αvβ6 positive cells was determined. Percentage of αvβ6 positive epithelium was determined as follows:

(Number positive stained squares/total number of squares)x100

### Human Tissue and Ethical Approval

Lung tissue from IPF patients (PF) was obtained either post mortem or from lung transplant patients following informed, written consent and ethical review (Ethical approval numbers: Nottingham Respiratory Research Unit 08/H0407/1; Papworth Hospital Research Tissue Bank, REC: 08/H0304/56; UCSF IRB #10–00198). In all cases the pathological diagnosis was usual interstitial pneumonia and the clinical diagnosis IPF was made based on ATS/ERS consensus criteria [22]. Non-fibrotic human lung tissue was obtained from non-cancerous tissue removed during surgery or from donor lungs unsuitable for transplant. All experiments were performed in accordance with the WMA Declaration of Helsinki.

### Statistics

All *in vitro* cell experiments were repeated a minimum of three separate times, with each experiment containing a minimum of two technical repeats. Data are expressed as mean data from the three independent experiments. Statistical significance was determined by two-tailed unpaired T test when comparing two data sets. A one-way ANOVA was used with a Dunnett’s multiple comparison post-test when comparing multiple data sets. All statistical analyses were performed using Graphpad Prism (Version 6). P < 0.05 was accepted as significant is all cases.

## Results

### αvβ6 integrin expression is mediated via TGFβ1 induced transcription of *ITGB6*

**Fig 1:**
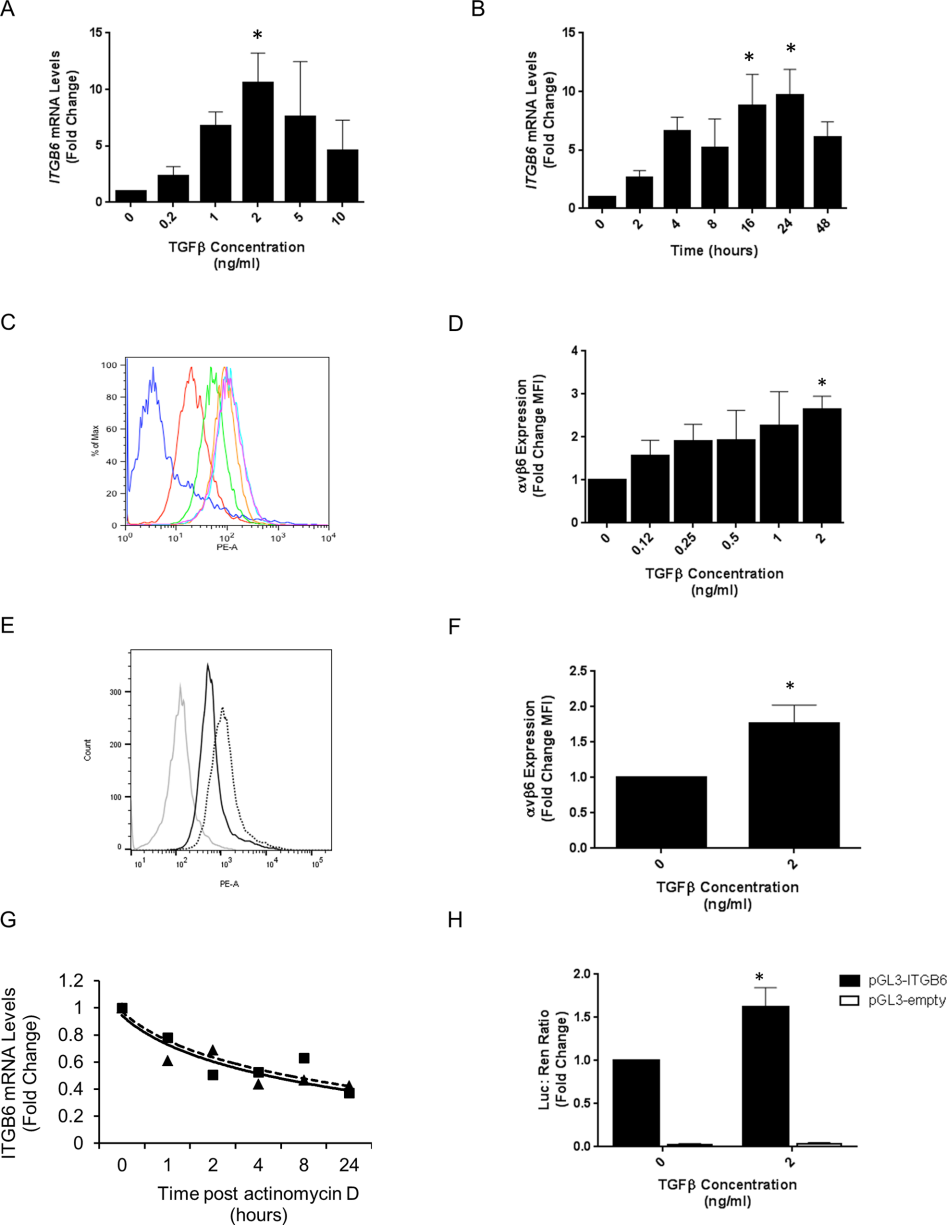
**A.** iHBECs were treated with increasing concentrations of TGFβ1 for 16 hours and *ITGB6* mRNA was measured by QPCR. Data are expressed as mean expression relative to control (0 ng/ml) ± SEM from three independent experiments. * p < 0.05 **B.** iHBECs were treated with 2 ng/ml TGFβ1 for over 48 hours and *ITGB6* mRNA was measured by QPCR at increasing time points. Data are expressed as mean expression relative to control (0 h) ± SEM from three independent experiments. * p < 0.05 **C.** iHBECs were treated with increasing concentrations of TGFβ1 (red = 0, green = 0.12, orange = 0.25, pink = 0.5, light blue = 1 and purple = 2 ng/ml. Dark blue = negative control) for 7 days and αvβ6 cell surface expression measured by flow cytometry. Experiment was repeated three times and histogram shows representative data from single experimental repeat. **D.** Amalgamated data from three independent experimental repeats of the experiment described in Fig 1C are demonstrated as a bar chart showing mean fold change in mean fluorescence intensity (MFI) ± SEM. * p < 0.05 **E.** iHBECs were treated with 0 or 2 ng/ml TGFβ1 for three days and αvβ6 integrin cell surface expression was measured by flow cytometry. Grey line = negative control, black line = 0 ng/ml TGFβ, black dotted line = 2 ng/ml TGFβ. Experiment was repeated three times and histogram shows representative data from single experimental repeat. **F.** iHBECs were treated with 2 ng/ml TGFβ1 for three days and αvβ6 integrin cell surface expression was measured by flow cytometry. Amalgamated data from three independent experiments are expressed as mean fold change in MFI ± SEM. * p < 0.05 **G.** iHBECs were treated with 0 or 2 ng/ml TGFβ1 for 4 hours followed by 5 μg/ml actinomycin D. *ITGB6* mRNA levels were then measured by QPCR at increasing time points up to 24 hours. Data are expressed as mean expression relative to control (0 h) ± SEM from three independent experiments. **H.** iHBECs transfected with either pGL3-*ITGB6* or empty pGL3 as a control were treated with 0 or 2 ng/ml TGFβ1 for 4 hours. Data are expressed as mean relative firefly / renilla luciferase ratio ± SEM from three independent experiments. * p < 0.05.

To confirm that TGFβ1 induced *ITGB6* gene transcription in iHBECs, cells were stimulated with increasing concentrations of TGFβ1 that resulted in increased *ITGB6* mRNA, which was maximal at 2ng/ml and decreased at higher concentrations (5 and 10ng/ml; Fig 1A). Furthermore, stimulation of iHBECs with 2ng/ml TGFβ1 caused a time dependent increase in *ITGB6* mRNA that was maximal between 16 and 24 hours (Fig 1B). Importantly, treatment of iHBECs with increasing concentrations of TGFβ1 for 7 days led to a concentration dependent-increase in cell surface expression of αvβ6 integrins that was maximal at 2 ng/ml (Fig 1C and 1D). Small increases in αvβ6 cell-surface expression in response to TGFβ1 stimulation were also observed after 3 days (Fig 1E and 1F) and small increases were observed as early as 24 hours after stimulation (data not shown).

To determine the mechanism of TGFβ1 induced *ITGB6* mRNA expression we utilised the inhibitor of transcription actinomycin D. We observed no difference in the decay rate of *ITGB6* mRNA following actinomycin D treatment between TGFβ1 treated (half-life = 5.9 hours) and untreated (half-life = 5.5 hours) iHBECs (Fig 1G) suggesting that TGFβ1 does not affect stability of *ITGB6* mRNA. To confirm that TGFβ1-induced *ITGB6* was via transcriptional activation of the *ITGB6* gene promoter we utilised a pGL3-*ITGB6* promoter luciferase reporter construct. Stimulation of iHBECs transfected with pGL3-*ITGB6* with TGFβ1 caused a significant increase in luciferase activity after 4 hours (Fig 1H).

### Basal Expression of αvβ6 Integrins is Mediated via Basal αvβ6-induced TGFβ Activation

**Fig 2:**
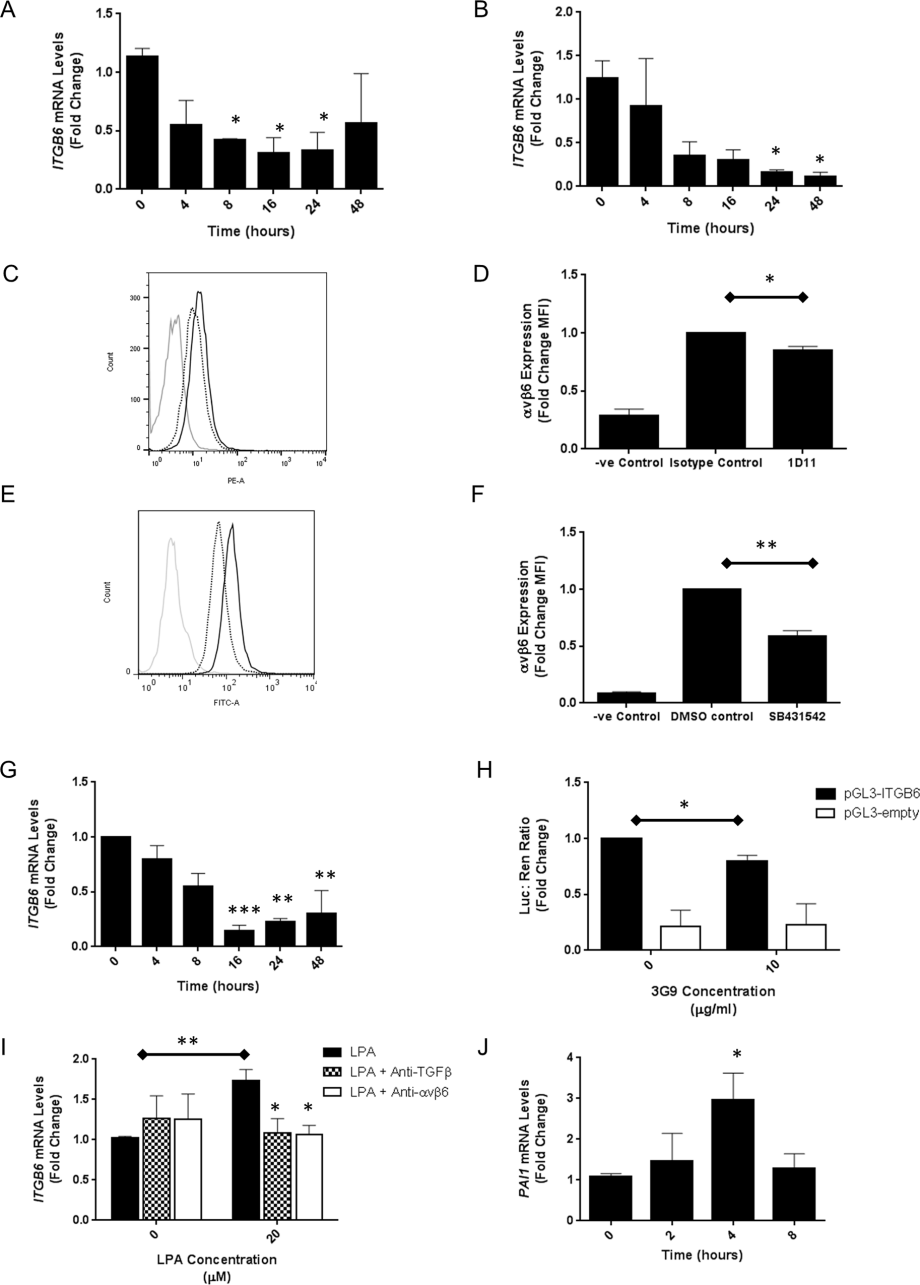
**A.** iHBECs were treated with 10 μg/ml anti-TGFβ and *ITGB6* mRNA was measured by QPCR at increasing time points. Data are expressed as mean expression relative to control (0 h) ± SEM from three independent experiments. * p < 0.05 **B.** iHBECs were treated with 10 μM Alk5 inhibitor and *ITGB6* mRNA was measured by QPCR at increasing time points. Data are expressed as mean expression relative to control (0 h) ± SEM from three independent experiments. * p < 0.05 **C.** iHBECs were treated with 10 μg/ml anti-TGFβ or an isotype control and αvβ6 cell surface expression was measured by flow cytometry after 3 days. Three independent experiments were performed and histogram shows representative data from one experimental repeat. Grey = negative control, black solid = 0 μg/ml, black dotted = 10 μg/ml. **D.** Amalgamated data from three independent experimental repeats of the experiment described in Fig 2C are demonstrated as a bar chart showing mean fold change in MFI ± SEM. * p < 0.05 **E.** iHBECs were treated with 0 or 10 μM Alk5 inhibitor (SB431542) and αvβ6 cell surface expression was measured by flow cytometry after 3 days. Three independent experiments were performed and histogram shows representative data from one experimental repeat. Grey = negative control, black solid = 0 μM, black dotted = 10 μM. **F.** Amalgamated data from three independent experimental repeats of the experiment described in Fig 2E are demonstrated as a bar chart showing mean fold change in MFI ± SEM. ** p < 0.01 **G.** iHBECs were treated with 0 or 10 μg/ml anti-αvβ6 and *ITGB6* mRNA was measured by QPCR at increasing time points. Data are expressed as mean expression relative to control (0 h) ± SEM from three independent experiments. ** p < 0.01, *** p < 0.005 **H.** iHBECs transfected with either pGL3-*ITGB6* or empty pGL3 as a control were treated with 0 or 10 μg/ml anti-αvβ6 for 4 hours. Data are expressed as mean relative firefly / renilla luciferase ratio ± SEM from three independent experiments. * p < 0.05 **I.** iHBECs were pre-treated with no antibody (black bars), 10 μg/ml anti-TGFβ (checked bars) or 10 μg/ml anti-αvβ6 (white bars) for one hour then stimulated with 20 μM LPA. *ITGB6* mRNA was measured by QPCR after 0 and 8 hours. Data are expressed as mean expression relative to control (0 h, no antibody) ± SEM from three independent experiments. * p < 0.05, ** p < 0.01 **J.** iHBECs were treated with 20μM LPA and *PAI* mRNA was measured by QPCR at 0, 2, 4 and 8 hours. Data are expressed as mean expression relative to control (0 h) ± SEM from three independent experiments. * p < 0.05.

It is known that αvβ6 integrins are a central mechanism through which TGFβ1 is activated, and we have shown that active TGFβ1 upregulates αvβ6 integrins through increased *ITGB6* transcription, consistent with the presence of an autocrine loop of αvβ6-mediated TGFβ1 activation induced *ITGB6* gene expression. Our data clearly show that αvβ6 integrins are expressed basally in the absence of exogenous TGFβ1 by iHBECs (Fig 1C). Therefore, we hypothesised that basal αvβ6 integrin expression observed in human epithelial cells would be dependent on active endogenous TGFβ1 generated by epithelial αvβ6 integrins. iHBECs were cultured in the presence of a pan-TGFβ blocking antibody or a small molecular inhibitor of TGFβ receptors (SB431542), both of which inhibited basal *ITGB6* mRNA in a time-dependent manner that was maximal at 24 hours and sustained over 48 hours (Fig 2A and 2B). Importantly, αvβ6 cell surface expression was significantly inhibited after 3 days treatment with either the pan-TGFβ blocking antibody (Fig 2C & 2D) or the Alk5 inhibitor SB431542 (Fig 2E & 2F). To investigate whether basal TGFβ1-dependent *ITGB6* and αvβ6 expression in lung epithelial cells was via αvβ6 integrin-mediated activation of TGFβ1 we used a blocking antibody directed against the αvβ6 integrin. The αvβ6 blocking antibody reduced *ITGB6* mRNA levels in iHBECs in a time-dependent manner, again maximal after 16 hours (Fig 2G), recapitulating the effect of both the pan-TGFβ blocking antibody and SB431542 in iHBECs. The reporter construct pGL3-*ITGB6*, or empty pGL3, were transfected into iHBECs in the presence or absence of the αvβ6 integrin blocking antibody 6.3G9 for 4 hours (Fig 2H). In the presence of the αvβ6 integrin-blocking antibody there was a small but statistically significant reduction in promoter activity demonstrating that basal αvβ6-mediated TGFβ1 activation regulates *ITGB6*.

We have previously demonstrated that LPA induces αvβ6 integrin mediated TGFβ1 activation in primary bronchial epithelial cells [10]. Therefore, we hypothesised that LPA stimulation of iHBECS would amplify *ITGB6* expression in a TGFβ1 dependent manner. LPA stimulation of iHBECS for 4 hours increased *ITGB6* mRNA, which was inhibited by both pan-TGFβ and αvβ6 blocking antibodies (Fig 2I). To determine whether LPA had induced TGFβ1 activity, PAI1 mRNA levels were measured and observed to be elevated (Fig 2J).

**Fig 3:**
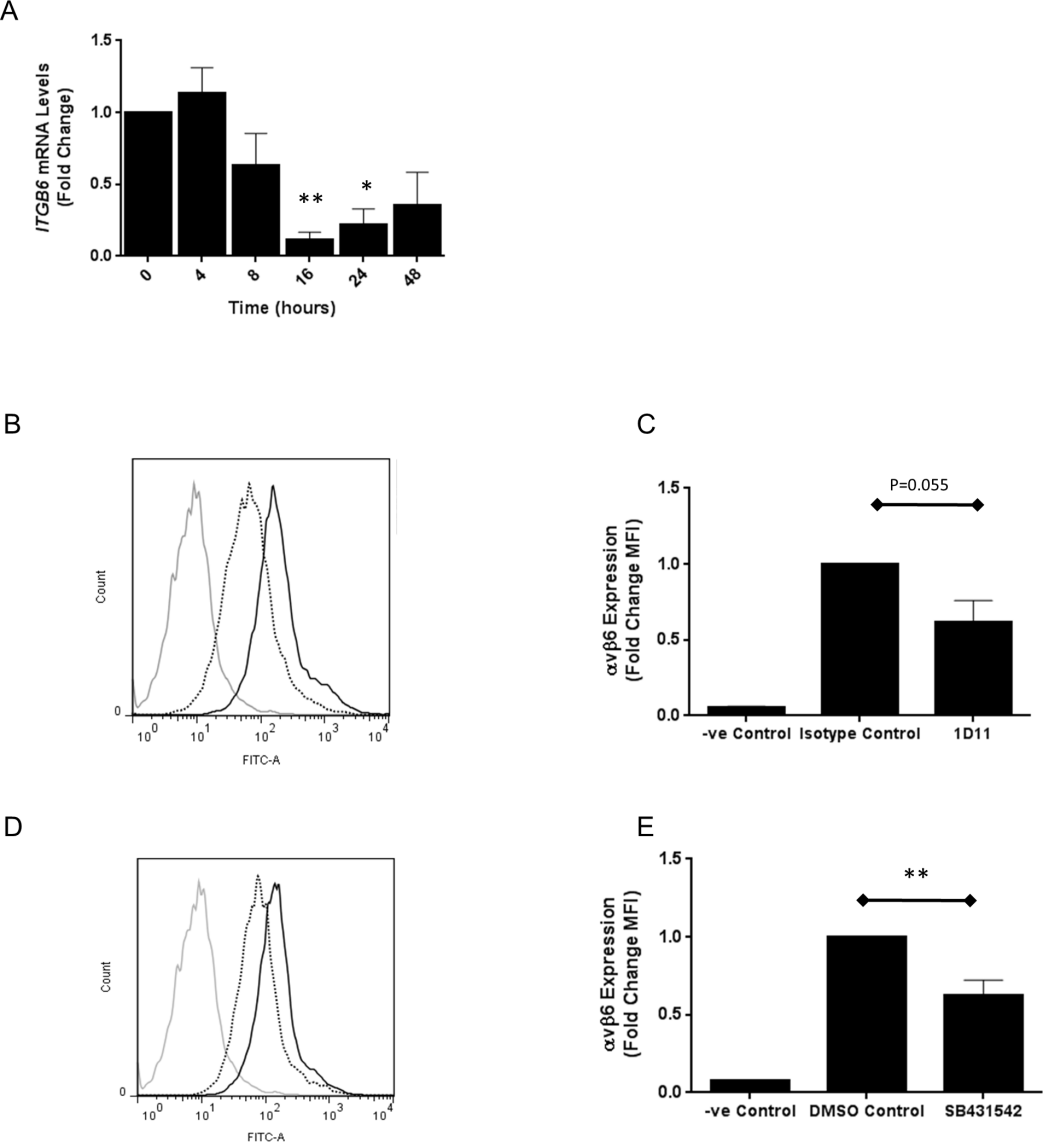
**A.** SAECs were treated with 10 μM Alk5 inhibitor (SB431542) and *ITGB6* mRNA was measured by QPCR at increasing time points. Data are expressed as mean expression relative to control (0 h) ± SEM from three independent experiments. * p < 0.05, ** p < 0.01 **B.** SAECS were treated with 10 μg/ml anti-TGFβ or an isotype control and αvβ6 cell surface expression measured by flow cytometry after three days. Three independent experiments were performed and the histogram shows representative data from one experimental repeat. Grey = negative control, black solid = isotype control, black dotted = anti-TGFβ. **C.** Amalgamated data from three independent experimental repeats of the experiment described in Fig 3B are demonstrated as a bar chart showing mean fold change in MFI ± SEM. **D.** SAECs were treated with 10 μM Alk5 inhibitor (SB431542), or an equivalent volume of the vehicle DMSO as a control, and αvβ6 cell surface expression measured by flow cytometry after three days. Three independent experiments were performed and the histogram shows representative data from one experimental repeat. Grey = negative control, black solid = DMSO control, black dotted = SB431542. **E.** Amalgamated data from three independent experimental repeats of the experiment described in Fig 3D are demonstrated as a bar chart showing mean fold change in MFI ± SEM. ** p < 0.01.

To confirm that TGFβ1 inhibition was sufficient to reduce basal *ITGB6* and αvβ6 expression in primary lung epithelial cells, small airway epithelial cells (SAECs; Lonza, UK) were used. Recapitulating the effect observed in iHBECs, a pan-TGFβ blocking antibody reduced *ITGB6* mRNA expression in a time dependent manner that was maximal at 16 hours (Fig 3A) and inhibited αvβ6 cell surface expression after 3 days of treatment (Fig 3B & 3C). Additionally, the Alk5 inhibitor SB431542 also reduced αvβ6 cell surface expression in SAECs after three days (Fig 3D & 3E).

### TGFβ1-induced *ITGB6* Gene Expression is mediated via Smad Signalling *in vitro*

**Fig 4:**
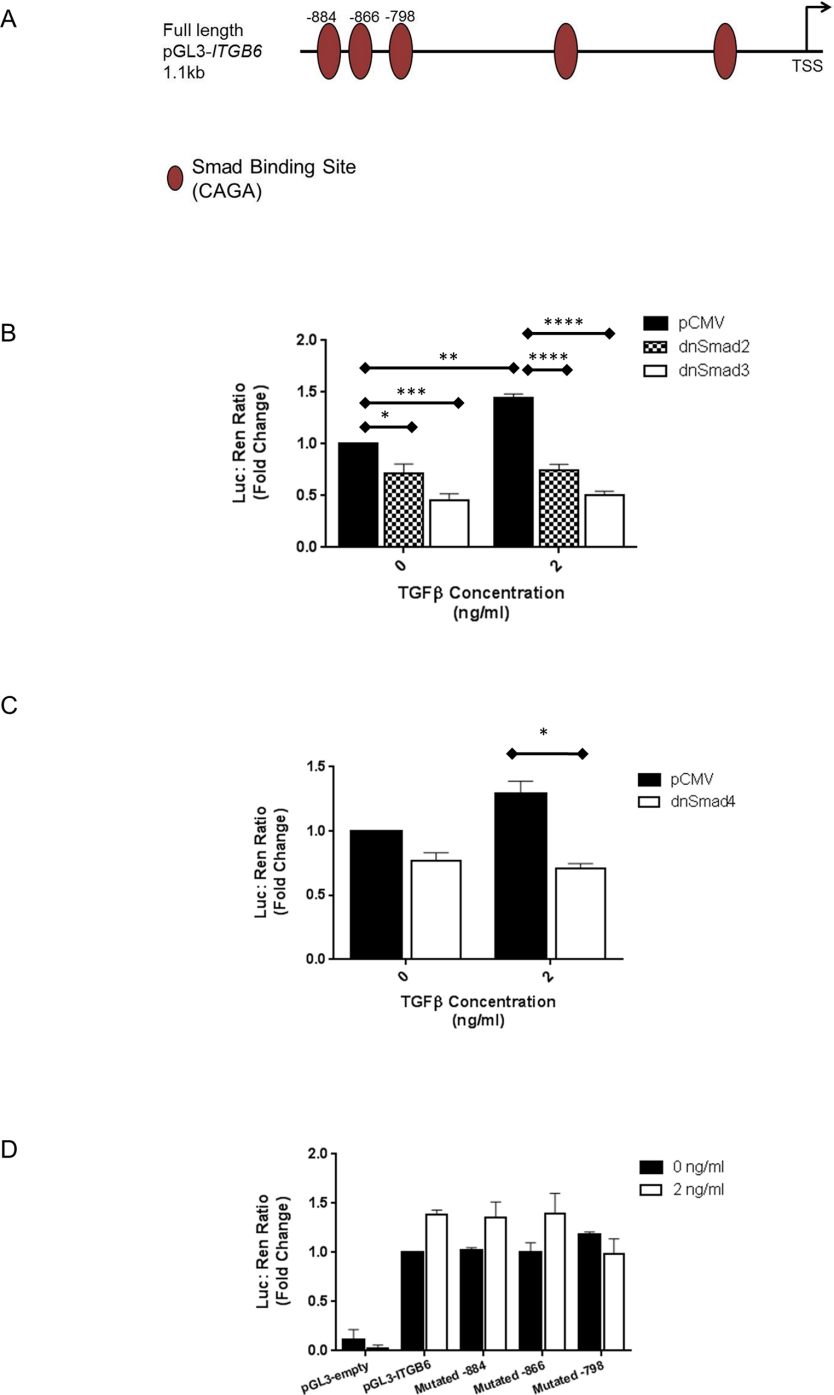
**A.** Schematic diagram showing the location of canonical Smad binding sites (CAGA) in the 1.1kb pGL3-*ITGB6* promoter reporter construct. **B.** iHBECs transfected with either empty pCMV vector, dnSmad2 or dnSmad3 together with the pGL3-*ITGB6* promoter reporter construct were treated with either 0 or 2 ng/ml TGFβ1 for 4 hours and luciferase activity measured. Data are expressed as mean fold change firefly / renilla luciferase (relative to pCMV, 0 ng/ml) ± SEM from three independent experiments. * p < 0.05, *** p < 0.005, **** p < 0.0001 **C.** iHBECs transfected with either empty pCMV vector or dnSmad4 together with the pGL3-*ITGB6* promoter reporter construct were treated with either 0 or 2 ng/ml TGFβ1 for 4 hours and luciferase activity measured. Data are expressed as mean fold change firefly / renilla luciferase (relative to pCMV, 0 ng/ml) ± SEM from three independent experiments. * p < 0.05 **D.** iHBECs were transfected with either the unmutated pGL3-*ITGB6* promoter reporter construct, or constructs containing mutations in key Smad binding sites at -884, -866 and -798 then treated with 0 or 2 ng/ml TGFβ1 for 4 hours. Luciferase activity was then measured. Data are expressed as mean fold change firefly / renilla luciferase (relative to pGL3-*ITGB6*, 0 ng/ml) ± SEM from three independent experiments.

Having identified that epithelial αvβ6 integrin expression is regulated by autocrine TGFβ1 activated *ITGB6* gene transcription we investigated the molecular mechanisms responsible. The *ITGB6* promoter region contained within pGL3-*ITGB6* contains 5 canonical Smad binding sites containing CAGA motifs (see Fig 4A). To determine whether canonical Smad signalling was involved we utilised dominant negative (dn) constructs directed towards Smad2, Smad3 and Smad4 and assessed their effect on basal and TGFβ1-induced *ITGB6* promoter activity. Both dnSmad2 and dnSmad3 constructs reduced basal and TGFβ1-induced *ITGB6* promoter activity, although the effect was considerably greater for dnSmad3 (Fig 4B). Furthermore, a dnSmad4 construct also inhibited both basal and TGFβ1-induced promoter activity (Fig 4C), consistent with the effects observed for dnSmad2 and dnSmad3. Importantly, disrupting the Smad binding site (CAGA) found at -798 from the transcription start site (TSS) in the promoter reporter construct driving the luciferase gene, using site directed mutagenesis, abrogated *ITGB6* transcriptional activity in response to TGFβ1 (Fig 4D).

**Fig 5:**
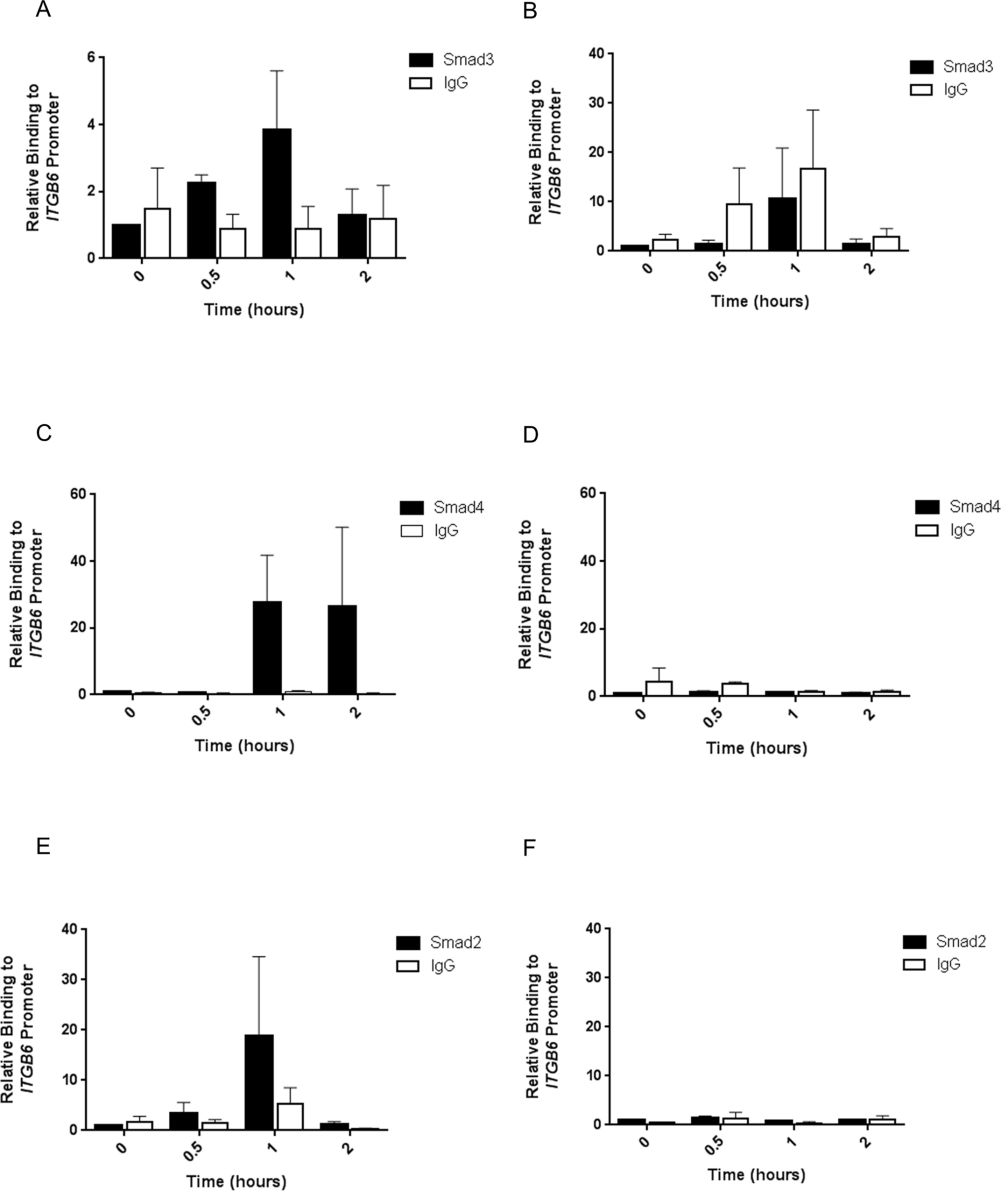
**A.** iHBECs were treated with 2 ng/ml TGFβ1 and binding of Smad3 to the *ITGB6* promoter at approximately -936 to -755 was assessed after 0, 0.5, 1 and 2 hours using ChIP. Data are expressed as mean relative binding (relative to 0 h) ± SEM from 3 independent experiments. **B**. iHBECs were treated with 2 ng/ml TGFβ1 and binding of Smad3 to the *ITGB6* promoter at approximately -1608 to -1500 was assessed after 0, 0.5, 1 and 2 hours using ChIP. Data are expressed as mean relative binding (relative to 0 h) ± SEM from 3 independent experiments. **C.** iHBECs were treated with 2 ng/ml TGFβ1 and binding of Smad4 to the *ITGB6* promoter at approximately -936 to -755 was assessed after 0, 0.5, 1 and 2 hours using ChIP. Data are expressed as mean relative binding (relative to 0 h) ± SEM from 3 independent experiments. **D.** iHBECs were treated with 2 ng/ml TGFβ1 and binding of Smad4 to the *ITGB6* promoter at approximately -1608 to -1500 was assessed after 0, 0.5, 1 and 2 hours using ChIP. Data are expressed as mean relative binding (relative to 0 h) ± SEM from 3 independent experiments. **E.** iHBECs were treated with 2 ng/ml TGFβ1 and binding of Smad2 to the *ITGB6* promoter at approximately -936 to -755 was assessed after 0, 0.5, 1 and 2 hours using ChIP. Data are expressed as mean relative binding (relative to 0 h) ± SEM from 3 independent experiments. **F.** iHBECs were treated with 2 ng/ml TGFβ1 and binding of Smad2 to the *ITGB6* promoter at approximately -1608 to -1500 was assessed after 0, 0.5, 1 and 2 hours using ChIP. Data are expressed as mean relative binding (relative to 0 h) ± SEM from 3 independent experiments.

We next sought to show that Smad proteins were capable of binding to the region of the *ITGB6* promoter around -798 from the TSS using ChIP assays. Both Smad3 and Smad4 bound to the *ITGB6* promoter 1 hour following TGFβ1 stimulation (Fig 5A and 5C), but no binding above IgG control levels for either Smad3 or Smad4 was detected at a control region of DNA approximately 1.6kb upstream of the TSS that did not contain Smad binding sites (Fig 5B and 5D). Smad2 did bind to the *ITGB6* promoter 1 hour after TGFβ1 stimulation, however, binding was more variable (Fig 5E). Importantly, Smad2 did not bind to the control, upstream region of DNA (Fig 5F).

### Smad3 Regulates αvβ6 Expression *in vivo*

**Fig 6:**
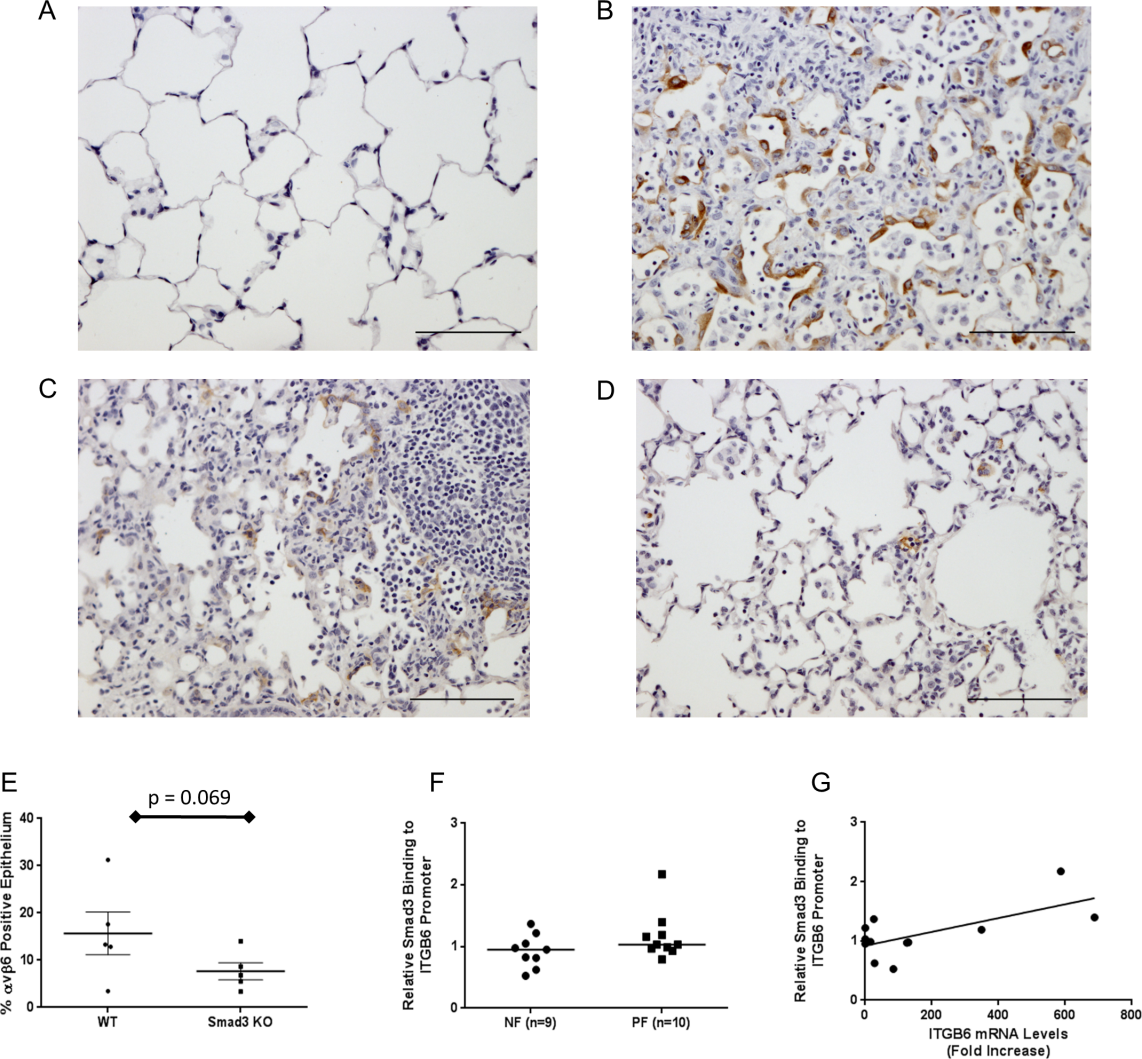
**A.** Rats were treated with a control adenovirus for 21 days and lung tissue was stained by immunohistochemistry for αvβ6 integrins. Figure is representative of n = 3 animals. **B**. Rats were treated with a TGFβ1 over-expression adenovirus for 21 days and lung tissue was stained by immunohistochemistry for αvβ6 integrins. Figure is representative of n = 3 animals. **C**. *Smad3*^*+/+*^ control animals were treated with a TGFβ1 over-expression adenovirus for 21 days and lung tissue was stained by immunohistochemistry for αvβ6 integrins. Figure is representative of n = 5 animals **D**. *Smad3*^*-/-*^ control animals were treated with a TGFβ1 over-expression adenovirus for 21 days and lung tissue was stained by immunohistochemistry for αvβ6 integrins. Figure is representative of n = 4 animals **E**. αvβ6 immunohistochemistry described in Fig 5C and 5D was quantified using in a blind manner using a semi-quantitative, user-dependent method. Data are expressed as % of αvβ6 positive alveolar epithelium. Bars show mean ± SD **F**. Human lung tissue from pulmonary fibrosis donors (n = 10) and non-fibrotic controls (n = 9) was subjected to ChIP analysis to determine basal levels of Smad3 binding to the endogenous *ITGB6* promoter in the region -936 to -755 from the transcription start site. Data are expressed as relative binding to the *ITGB6* promoter (relative to IgG levels in each donor sample) and the median shown. Relative binding of 1 or below demonstrates no binding of Smad3 above IgG control levels. **G**. Relative binding of Smad3 measured in Fig 5F was correlated with measured levels of *ITGB6* mRNA in each donor sample using linear regression analysis. R^2^ = 0.44, p = 0.019.

To investigate this pathway *in vivo*, active TGFβ1 was over-expressed in rats using an adenoviral system. Rats treated with a control adenovirus displayed normal alveolar structure and some evidence of low-level αvβ6 expression (Fig 6A). In contrast, rats treated with an adenovirus encoding active TGFβ1 developed extensive pulmonary fibrosis associated with increased expression of αvβ6 integrins within the alveolar epithelium (Fig 6B). To confirm a role for Smad3 in mediating TGFβ1-induced increases in αvβ6 expression *in vivo*, *Smad3*^*-/-*^ and wild-type control mice were treated with adenoviral active TGFβ1. Extensive αvβ6 immunostaining was evident in the alveolar epithelium around regions of fibrosis in wild-type mice treated with adenoviral TGFβ1 (Fig 6C). In comparison, *Smad3*^*-/-*^ mice exposed to adenoviral TGFβ1 had reduced levels of αvβ6 integrin within the lung parenchyma (Fig 6D). The percentage of the alveolar epithelium expressing αvβ6 was quantified and a trend towards reduced expression in the Smad3^-/-^ animals was observed (Fig 6E).

Finally, we investigated whether binding of Smad3 to the *ITGB6* promoter was aberrant in IPF in human lung tissue using chromatin immunoprecipitation. Binding of Smad3 to the *ITGB6* promoter was detected in all donors samples tested (Fig 6F). We found no significant difference in binding levels of Smad3 to the *ITGB6* promoter between IPF (n = 10) and non-fibrotic control (n = 9) donors, although one IPF donor did demonstrate markedly increased Smad3 binding above all other donors (Fig 6F). When binding of Smad3 was correlated with *ITGB6* mRNA levels by linear regression a moderate, but significant (p = 0.02), positive correlation was observed.

## Discussion

αvβ6 integrin-mediated activation of the pro-fibrotic cytokine TGFβ1 is a key process during fibrogenesis in organs such as the lungs and kidneys [6, 9]. Expression of αvβ6 integrins is both tightly regulated, and restricted to epithelial cells, but is dramatically upregulated in response to injury in both fibrotic lung disease and in experimental models of pulmonary fibrosis [6, 10, 13]. The molecular mechanisms governing increased αvβ6 expression have not been fully delineated although early reports suggested a role for TGFβ1 [16] and, more recently, both the ets-domain containing protein Elk1 and the glucocorticoid receptor have been implicated [20]. In the present study we have confirmed the autocrine loop of αvβ6-mediated TGFβ1 activation regulating αvβ6 integrins originally suggested by Araya and colleagues in 2007 [17] and delineated the molecular pathways governing TGFβ1-induced *ITGB6/*αvβ6 both *in vitro* and *in vivo*.

Our data demonstrate that TGFβ1 upregulates αvβ6 integrin cell surface expression and *ITGB6* mRNA expression in human lung epithelial cells, supporting similar observations described previously in guinea pig epithelial cells [16]. Importantly, we build on these observations by demonstrating for the first time TGFβ1-induced *ITGB6*, and αvβ6 integrin, expression is mediated through Smad-dependent transcriptional upregulation of the *ITGB6* promoter. Importantly, we confirm a role for Smad signalling *in vivo* using a TGFβ1-overexpression model of pulmonary fibrosis.

Our data using the inhibitor of transcription actinomycin D suggests that *ITGB6* mRNA is very stable with a half-life of greater than 24 hours. This would explain the progressive accumulation of *ITGB6* mRNA over 24 hours following TGFβ1 stimulation that we, and others, have observed [16]. In contrast these data show that TGFβ1 stimulation of the exogenous *ITGB6* promoter-luciferase reporter construct leads to considerably lower increases in promoter activity than observed from the endogenous promoter at a similar time. Extra-chromosomally located promoter constructs are not subject to cis- and trans-activating regulation that may affect the overall level of gene expression. Furthermore, luciferase has a considerably shorter half-life (3 hours) than the endogenous *ITGB6* gene. Thus, the different magnitude of response between the luciferase reporter construct and the endogenous *ITGB6* gene to TGFβ1 invites us to speculate that targeting the 5’- or 3’- untranslated regions may be a useful strategy to limit TGFβ1-induced effects on *ITGB6*. Indeed *ITGB6* mRNA is predicted to be a target for mir19a (http://www.ncrna.org/glocal/), which is down regulated in fibrosis in the liver [23, 24], heart [25] and lung [26].

Smad3 may be a central TGFβ1 signalling intermediate in the pathogenesis of pulmonary fibrosis. Smad3 induces fibroblast-to-myofibroblast transdifferentiation [27] and *Smad3*^*-/-*^ mice are protected from bleomycin-induced lung fibrosis [28]. We show that inhibition of Smad3 *in vitro* reduced *ITGB6* gene activity. A role for Smad2 can’t be completely excluded as inhibition of Smad2 with a dominant negative construct had a negative effect on basal *ITGB6* expression, however, it was smaller than observed with the dominant negative Smad3 construct. Importantly, the dominant negative co-Smad, Smad4, reduced both basal and TGFβ1-induced *ITGB6* promoter activity recapitulating the effects of Smad3. Fundamentally, we show that loss of Smad3 *in vivo* interrupts TGFβ1-induced upregulation of αvβ6 integrins within the lung epithelium, confirming the *in vitro* data. While it is not clear that the lack of fibrogenesis following TGFβ1 over-expression is the direct result of reduced αvβ6 integrin expression, the data nonetheless confirm that Smad3 is required for TGFβ1-induced upregulation of αvβ6 integrins *in vivo*.

Using human lung tissue obtained from IPF patients and non-fibrotic controls we investigated whether Smad3 signalling was aberrant in human disease. We used chromatin immunoprecipitation to assess direct binding of Smad3 to the *ITGB6* promoter but we found no convincing evidence that Smad3 binding was disrupted in PF samples, at least in advanced, end-stage disease. It is possible that this is a kinetic issue and explained by the fact that the tissue was collected from patients with advanced, end-stage disease. Tissue collected earlier in the pathogenic process may demonstrate increased binding of Smad3 to the *ITGB6* promoter, however obtaining human lung tissue from IPF patients with newly diagnosed IPF is problematic. One patient sample demonstrated high levels of Smad3 binding to the *ITGB6* promoter compared with other donor tissue tested, raising the possibility that aberrant Smad3 binding could be one of multiple possible molecular defects that contribute to variations in αvβ6 expression in IPF, and highlights the potential for heterogeneity in the molecular mechanisms driving disease in IPF patients. We have recently identified a separate defect that may also contribute enhanced αvβ6 integrin expression in IPF [20].

The strengths of this study include the use of both small molecular inhibitors and antibodies to confirm the effect of TGFβ1 in regulation αvβ6 integrin expression in human cells, validating results discovered in primary guinea pig epithelial cells [16]. This strategy ensures that the data described are both repeatable in multiple systems and robust. Similarly confirming the results using dominant negative constructs and genetically manipulated animals demonstrates replication between systems and highlights the in vivo relevance of the observations. Ultimately these data use a range of techniques and a number of experimental systems to determine the mechanism of a key molecular pathway of central relevance to pulmonary fibrosis.

There are also some weaknesses associated with our approach. These include the use of extrachromosomally located reporters described earlier. This study also used *in vitro* genetic manipulation of Smad pathway molecules, which required the use of transient transfection techniques that have both variable transfection efficiency and time-limited effects on gene expression. Thus it is not possible to assess the effect on dominant negative Smad transfection on global levels of epithelial αvβ6 integrin expression, which our data suggest has a half-life of approximately 72 hours and would require cellular transfection efficiency well above 50%. Similarly, the use of ChIP assays to investigate binding of Smad proteins to the *ITGB6* promoter in both cultured epithelial cells and human lung tissue, is particularly prone to variations in amplitude making amalgamated data unsuitable for statistical analysis. This is due to variable formaldehyde fixation and inefficiency of the antibody to completely immunoprecipitate all the available antigen [29]. Therefore, ChIP assay results are considered qualitative rather than quantitative. Thus we have drawn conclusions regarding the role of Smad-mediated regulation of *ITGB6* using binding data from ChIP assays in combination with data from experiments using site directed mutagenesis and dominant negative constructs.

In summary, these studies identify an important role for Smad3 in regulating αvβ6 integrins and the potential for dysregulation of this pathway to impact on pulmonary fibrosis. Future studies will be aimed at determining the role of Smad3 regulation of *ITGB6* gene expression in epithelial cells obtained from patients with interstitial lung disease, as well as understanding how changes in Smad3 function and regulation during wound repair may lead to dysregulated *ITGB*6 gene expression. In conclusion, this study elucidates the homeostatic signalling pathways governing TGFβ1-induced *ITGB6* and αvβ6 integrin expression in the human lung, confirms previous suggestions of an autocrine of αvβ6-mediated TGFβ1 activation regulating αvβ6 expression and illustrates a potential role for dysregulation of this pathway in fibrogenesis.
